# A human rights assessment of a large mental hospital in Kenya

**DOI:** 10.11604/pamj.2021.40.199.30470

**Published:** 2021-12-01

**Authors:** Joy Muhia, Florence Jaguga, Victoria Wamukhoma, Jacqueline Aloo, Simon Njuguna

**Affiliations:** 1 Division of Mental Health, Ministry of Health, Nairobi, Kenya,; 2 Department of Mental Health, Moi Teaching and Referral Hospital, Eldoret, Kenya,; 3 Mathari National Teaching and Referral Hospital, Nairobi, Kenya

**Keywords:** Mental health, human rights, QualityRights, Convention on the Rights of Persons with Disabilities, Kenya

## Abstract

**Introduction:**

globally, human rights violations of persons with mental health conditions are rampant, and the quality of mental health services below that for general health services. The aim of this paper is to document the findings of an assessment of the quality of mental health services at the largest mental hospital in Kenya, and offer recommendations useful for service transformation.

**Methods:**

this was a cross-sectional study. Assessment was conducted guided by the World Health Organization (WHO) QualityRights Tool Kit, which assesses for compliance with five human rights themes drawn from the Convention on the Rights of People with Disabilities. Trained assessors collected data through document review, observation, and interviews with hospital staff and service users at Mathari National Teaching and Referral Hospital. The sample was composed of 64 interviewees.

**Results:**

overall, the facility was scored as “achievement initiated” indicating that there was evidence that steps had been taken to fulfil the five human rights themes but significant improvements were necessary. Five key gaps emerged: 1) the buildings and infrastructure were in a state of disrepair; 2) staffing was inadequate; 3) patients had no right to legal capacity; 4) there was gross neglect of patients as well as physical and verbal abuse; 5) there were no strategies in place to support community reintegration and independent living.

**Conclusion:**

significant improvements to infrastructure, staffing, and the quality of services are needed before the Mathari National Teaching and Referral Hospital meets the requirements of the Convention on the Rights of People with Disabilities.

## Introduction

Mental health services around the world have historically been characterized by human rights violations [1]. Legislation that allows coercive treatment, practices that perpetuate exclusion from employment, and physical abuse, exemplify some of the harsh treatments that persons with mental health conditions have been forced to endure [2]. Despite this, fewer than 50% of countries globally have bodies in existence that inspect mental health facilities for their compliance with human rights [3]. Low resource allocation to mental health further complicates these challenges particularly in Africa where the median government mental health expenditure per capita was estimated at only US$ 0.1 in 2017 [4]. In Kenya the quality of mental health services is concerning. Firstly, media reports have indicated rampant incidents of physical and verbal abuse within mental health facilities. Secondly, most mental health facilities have dilapidated infrastructure, unsanitary conditions and are overcrowded. Thirdly, the Mental Health Act 1989 provides for involuntary treatment and does not contain provisions that guarantee service users their right to legal capacity. Moreover, human and financial resource allocation to mental health is grossly inadequate. In 2017, less than 1% of the health budget was allocated to mental health. Kenya has a psychiatrist to population ratio of 1: 500,000 [4].

To address these challenges, the World Health Organization (WHO) launched the QualityRights (QR) movement in 2012 with the aim of transforming mental health services around the world and promoting the rights of persons with mental health conditions and psychosocial disabilities [5]. The initiative which seeks to align mental health services with the rights enshrined in the Convention on the Rights of Persons with Disabilities (CRPD), is gaining traction around the world. Several high- and low-income countries around the world have rolled out interventions aimed at fulfilling the objectives of the initiative [6,7]. Kenya has not been left behind and its Ministry of Health (MOH) launched the QualityRights initiative in 2019. The objectives of the initiative were set and are currently being implemented in collaboration with key stakeholders such as the Kenya National Commission on Human Rights, persons with lived experience, and Non-Governmental Organisations (NGOs) involved in mental health activities.

One of the key targets of the initiative was to conduct assessments and report on the quality of care and observance of human rights in the national referral mental health hospital, and in the 15 mental health units and 29 mental health outpatients' clinics throughout the country [8,9]. The first facility to be assessed was the Mathari National Teaching and Referral Hospital (MNTRH), a specialized referral facility for mental health patients. The aim of this paper is to outline the findings of an assessment of the quality of services at that facility, and provide recommendations for improvement. Prior audits by government agencies have focused on examining resources within the facility and have highlighted the limited budgetary allocation to the hospital, dilapidated infrastructure and severe staff shortages among other challenges [10]. The current report builds upon these previous reports by describing an assessment of the hospital for compliance with human rights standards enshrined in the CRPD.

## Methods

**Study design:** this was a cross-sectional study.

**Study setting:** the study was conducted at Mathari National Teaching and Referral Hospital. The hospital is the largest mental health facility in Kenya. It was started as a small pox isolation centre in 1904 and later transformed into a mental facility [11]. The hospital functions as a treatment, research and training centre. In addition, the facility has a maximum-security section that was opened in 1978 and caters for law offenders with mental health conditions. The hospital has a bed capacity of 700, with 332 in the civil section (for non-offenders) and 377 in the Maximum-Security Unit. The civil section is organized into 9 wards and 3 out-patient clinics. The hospital has bed occupancy of 119% in the civil unit and 115% in the Maximum-Security Unit. The facility which has a total of 386 staff, admits adults aged above 18 years only.

**Study population and sampling:** the study targeted staff and service users who were at the Mathari National Teaching and Referral Hospital during the assessments. For purposes of the assessment, the facility was organised into 4 zones (Table 1). We divided the maximum security unit into 3 sections to ensure thorough assessment. Each zone was assigned to a team of 3 assessors. The assessing committee made the decision to interview 2-4 staff and 2-4 service users per section given the time allocated for the assessment (2 days) in relation to the size of the hospital. The assessors additionally felt that such numbers would give sufficient information because the interviews would be augmented by observation and document review. In each ward/clinic, the assessors identified service users that appeared stable with no acute psychiatric symptoms. The staffs were not involved in the process of selection of service users to be interviewed in order to avoid identifying patients that they knew might give a desirable response. The assessors additionally identified staff that were on duty at the time of the assessment and were willing to participate in the interviews. All identified staff and service users were explained to the nature of the assessment, assured of confidentiality, and verbal consent obtained before the interviews. Those who did not consent to the interviews were excluded. In total 35 staff and 42 service users were interviewed (Table 1).

**Table 1:** assessment zones and number of participants interviewed

Zone	Sections	Number of staff interviewed	Number of service users interviewed
**A**	Psychiatry outpatient clinic	2	3
	Ward 2 (female)	2	2
	Ward 6 (male)	2	2
	Maximum Security Unit (MSU) section A	3	4
**B**	Ward 5 (female)	2	2
	Maximum Security Unit (MSU) section B	3	4
	Ward 9 (male)	2	3
	Kitchen	2	2
**C**	Ward 6 female	2	3
	Ward 8 male	2	3
	Maximum Security Unit (MSU) section C	3	4
	Substance use disorder treatment unit	2	2
**D**	Methadone clinic	2	2
	Amenity	2	2
	Ward 5 (male)	2	2
	Forensic clinic	2	2

**Data collection tool:** the WHO QualityRights Assessment Tool Kit (4) was used to collect data. The tool contains questions that assess for compliance of a mental health facility with rights drawn from the CRPD. The questions are organized into 5 themes that have been organized into standards and criteria. The 5 themes are as follows: (i) theme 1: the right to an adequate standard of living (article 28); (ii) theme 2: the right to enjoyment of the highest attainable standard of physical and mental health (article 25); (iii) theme 3: the right to exercise legal capacity and the right to personal liberty and security of person (articles 12 and 14); legal capacity refers to the right to hold rights and the right to exercise those rights; legal capacity is an inherent and inalienable right [12]; (iv) theme 4: freedom from torture or cruel, inhuman or degrading treatment or punishment and from exploitation, violence and abuse (articles 15 and 16); (v) theme 5: the right to live independently and be included in the community (article 19). Each theme, standard and criterion is scored as follows: “achieved in full ” (there is evidence that the criterion, standard or theme has been fully realized); “achieved partially” (there is evidence that the criterion, standard or theme has been fully realized but some improvement is necessary); “achievement initiated” (there is evidence that steps have been taken to fulfil the criterion, standard or theme, but significant improvement is necessary); “not initiated ” (there is no evidence or attempts or steps towards fulfilling the criterion, standard or theme); “not applicable” (the criterion, standard or theme does not apply to the facility in question).

**Preparation for the assessment:** a team of 12 assessors were selected from a cohort of participants who had completed an e-training and attended a 5-day face to face training on QualityRights. Prior to the exercise, the selected assessors underwent a 4-day training on how to assess mental health facilities using the WHO QualityRights Tool Kit (4). This latter training included a mock assessment of the mental ward at the Gilgil Sub-County Hospital, one of the oldest mental health facilities in Kenya.

**Assessment procedure:** administrative approval to conduct the assessment was sought from the Kenyan Ministry of Health (MOH) and the medical superintendent in-charge of the MNTRH. On the day of the assessment a courtesy call was made to the facility medical superintendent who reiterated his support for the assessment and introduced the assessing team to the ward nurse managers. The assessments were conducted by a team comprised of human rights lawyers and activists, representatives from service user support groups (persons with lived experience), members of mental health advocacy groups, representatives from non-governmental organizations (NGOs) involved in mental health activities, and mental health professionals (psychiatrists, psychologists, nurses and occupational therapists). Interviews were conducted with staff and service users in a private space within the wards or clinics. Documents reviewed included service user´s medical records and hospital policies. Observation in each ward was done both during the day and at night. The exercise was conducted between 16^th^ and 18^th^ October 2019. Each team of assessors took notes during the interviews, observation and review of records.

**Data collation and presentation:** a day after the assessment was completed, the assessors reviewed notes taken during the assessments, thoroughly discussed the findings, and built consensus on the scores and descriptions for each criterion, standard and theme. The team additionally discussed and built consensus on the overall score and its description. The findings of the assessments have been presented here as a narrative form.

**Ethical considerations:** ethical approval to publish the findings of the assessment was obtained from the Institutional Research Ethics Committee (IREC) of Moi University and Moi Teaching and Referral hospital (MTRH). Ethical approval was obtained after the reseach team confirmed that potentially identifying staff information e.g. cadre, gender would be excluded from this publication and that the research work presented no more than minimal risk to the participants.

## Results

**Overall score:** overall, the facility was scored as “achievement initiated” indicating that there was evidence that steps had been taken to fulfil the 5 human rights themes but significant improvements were necessary.

**The right to an adequate standard of living:** this theme was scored as “achievement initiated (A/I)”. The buildings were dilapidated and in a general state of disrepair. Even though the male and female quarters were separate, sleeping conditions were generally poor with overcrowding and insufficient bedding, and the sanitary requirements were inadequate and unclean. Food was balanced and water safe, but meal times were not conducive. For example supper was served at 3 p.m., 3 hours after lunch. Hospital uniform was provided but was worn out, ill-fitting and resembled the prison uniform in Kenya. The hospital had provided phones to allow service users to contact their relatives. The phones were however in the custody of the nurse in-charges and service users had to request to call. Privacy was not allowed during phone calls. Free movement around the facility was restricted.

**The right to enjoyment of the highest attainable standard of physical and mental health:** this theme was the highest scored at “achieved partially”. Services at the facility were accessible to all who needed without any discrimination. Psychotropic medication was affordable and was being used appropriately but stock-outs were frequent. General health services were adequate and were given without coercion. Skilled staff were however inadequate in number. The hospital had only 11 psychiatrists, 104 nurses, 3 nutritionists, 3 occupational therapists and no psychologist to cater to the 800 in-patients and 1000 out-patients seen at the facility daily. The staff had no training on rights of persons with mental conditions and psychosocial disabilities. There were no clear individualized recovery plans driven by the service user and there was little evidence of service user linkage to community support networks.

**The right to exercise legal capacity and the right to personal liberty and security of person:** this theme was scored as “achievement initiated” since some attempts to uphold the legal capacity of persons with mental conditions and psychosocial disabilities had been initiated. Service users had the right to confidentiality and access to their personal health information. However, their preferences were not always the priority for decisions on their treatment and recovery plans. In addition, there were no procedures and safeguards in place to prevent detention and treatment without free and informed consent. Moreover, substitute decision making was the main strategy for addressing impaired decision-making capacity.

**Freedom from torture or cruel, inhuman, or degrading treatment or punishment and from exploitation, violence and abuse:** this theme was scored as “not initiated”. There was no evidence that steps towards fulfilling this right had been taken. The service users in the facility particularly in the Maximum-Security Unit were exposed to cruel and inhuman conditions for example lack of sanitary facilities. Service users throughout the hospital were exposed to verbal, mental and physical abuse as well as physical and emotional neglect yet the facility had no mechanisms in place for reporting complaints. Alternative methods were not available for use in place of seclusion and restraint as means of de-escalating potential crises.

**The right to live independently and be included in the community:** this theme was scored as “not initiated”. Service users were not supported in gaining housing and financial resources necessary to live in the community. There were minimal efforts to help them access education and employment opportunities. The right of service users to participate in political and public life as well as engage in social, cultural, religious and leisure activities was not supported.

## Discussion

This is the first paper to report on the human rights assessment of a mental hospital in Kenya using the WHO QualityRights Tool Kit. Overall, the findings indicate that while the MNTRH had initiated attempts towards complying with human rights, there were significant improvements to be made. Five important gaps were identified. These are discussed below together with recommendations for policy and practice.

**1) Dilapidated infrastructure:** our assessment revealed that the hospital was dilapidated. This was also supported by the findings of the mental health taskforce report [13]. A similar state of disrepair was seen in mental health facilities in Egypt and Somalia [14,15]. In many African countries, the low budgetary allocation to mental health is a likely cause of poor infrastructure. A WHO report found that in 2017, the median expenditure for mental health per capita was lowest for the African region at US$ 0.1 [4]. The poor infrastructure at the MNTRH is a result of the dwindling budgetary allocation to the hospital despite increases in numbers of users seen over the years. For example in the year 2018/2019, the hospital´s recurrent allocation was reduced from 114 million to 92 million and drug allocation from 17 million to 2.6 million [16]. Additionally, during the financial year 2017/2018 the hospitals development fund was reduced from 75 to 18 million but none of the funds had been disbursed by the need of that financial year [10]. Fortunately, in 2020 the facility was gazetted as a Semi-Autonomous Government Agency (SAGA) and this was accompanied by a substantial increase in its budget [17].

**2) Inadequate staffing:** the problem of inadequate mental health staffing at the facility reflects an overall scarce mental health workforce throughout the country particularly within the public sector. Based on the MOH ideal ratios for psychiatrists (1: 30,000), psychiatric nurses (1: 6,000) and psychologists (1: 15,000), there is a current shortfall of over 1400 psychiatrists, over 7,000 psychiatric nurses and about 3,000 psychologists. Further, a majority of the trained mental health professionals work outside of the public sector. For instance, out of a total of 92 psychiatrists and 427 psychiatric nurses working in Kenya, only 36 (39%) and 187 (44%) respectively work within the public sector.

**3) Patients had no right to legal capacity:** the facility had not put in place procedures and safeguards to prevent detention and treatment without free and informed consent. This is consistent with occurrences in other low and middle income countries (LMICs). In Somalia, patients come to hospital chained and are not consulted about their treatment. In addition, no support agencies are available [15]. In Tunisia service users report that their will and preferences are not taken seriously by doctors. Strategies such as supported decision-making and advance directives have been reported as useful in ensuring that the will and preferences of persons with mental health conditions are upheld [18]. This is yet to be put in place in Kenya. Other LMICs have legislated measures to support legal capacity. The Indian mental health act contains provisions that support advance directives. Studies conducted so far have indicated feasibility and acceptability of advance directives among both service users and mental health workers in India, offering hope that such measures can be implemented in a low and middle income setting like ours [19]. The Kenyan Mental Health Act 1989 does not support supported decision making.

**4) Gross neglect of patients and physical and verbal abuse was rampant:** service users at MNTRH were exposed to unsanitary conditions as well as to verbal and physical abuse. Moreover, mechanisms for delivering and resolving complaints were non-existent. This problem seems to be a recurrent one with a previous report by the Kenya National Commission on Human rights (KNCHR) reporting rampant abuse in mental health settings in Kenya [20]. Physical and verbal abuse as well as neglect of persons with mental conditions can have serious negative consequences including physical and psychological harm [2]. Moreover, such abuse violates other fundamental rights such as the right to health. Urgent action is therefore required to address this problem.

**5) Violation of right to independent living and community involvement:** interventions that facilitate community reintegration of service users had not been incorporated into routine care, violating the right to independent living and community involvement. This reflects a mental health care system in Kenya that largely focuses on medical treatment and institutional care to the exclusion of community-based care and psychosocial interventions, approaches that are integral to recovery.

**Recommendations for improving the quality of services at MNTRH:** as a first next step, we propose that the MNTRH management together with the QR implementers from the MOH prepare a transformation plan for improving the quality of services at the facility. Strategies that should be implemented include: (i) short term improvements to infrastructure such as painting, purchase of mattresses, bedding, service user uniform, lighting fixtures. In the long term, the facility needs to plan for major renovations that should include fitting of sanitary facilities within the maximum-security unit; (ii) adjusting meal times to reflect those practiced within the local context; (iii) building capacity of staff on QualityRights to change attitudes and practice with the aim of ending violence and abuse; (iv) training staff on alternative methods of de-escalation in order to end coercive practices such as seclusion and restraint; (v) putting in place mechanisms through which complaints by service users may be addressed; (vi) in order to support independent living, MNTRH should collaborate with Non-Governmental Organisations (NGOs) that offer psychosocial support within Nairobi.

**Systems level recommendations:** the challenges identified at MNTRH are a reflection of an under resourced mental health system. We propose the following systems level strategies to address the gaps: (i) the government ought to increase the number of trained mental health professionals by offering incentives to enhance enrolment into mental health oriented courses and to encourage recruitment and retention of within the public sector; (ii) to facilitate independent living and social inclusion, the government should embark on a deinstitutionalization process that will entail the downsizing of psychiatric hospitals like MNTRH and establishment of community based mental health care services. In addition the government should put in place housing and employment support systems as well as meaningfully collaborate with NGOs to support activities that promote community inclusion; (iii) the Kenyan Mental Health Act 1989 needs to be amended to incorporate provisions that guarantee persons with disability their right to legal capacity; (iv) the government ought to put in place mechanisms to ensure effective independent monitoring of mental health facilities.

**Limitations:** the assessment used a small number of staff and service users due to time limitations and did not adhere to the numbers recommended in the WHO QualityRights Tool Kit. The findings here may therefore not be fully representative of the broad range of service users and staff at the facility. In addition the service users may have been reluctant to reveal negative information about the service. The interviews were however augmented by the assessors´ observations conducted both during the day and night, as well as document review. The findings are therefore useful for policy and practice of mental health in Kenya.

## Conclusion

The assessment of MNTRH revealed that the facility had taken steps towards complying with the rights enshrined in the CRPD. However, major gaps emerged. The infrastructure was in a state of disrepair, service users were exposed to abuse and coercion, and there were no services to support independent living. Significant improvements are required before the MNTRH meets the requirements of the CRPD.

**Funding:** financial support to conduct the assessment was received from Kenya National Commission on Human Rights (KNCHR). No funding was received to assist with the preparation of this manuscript.

### What is known about this topic

- Mental health services are characterized by human rights violations like coercion, involuntary treatment, deprivation of liberty and legal capacity;
- Mental health facilities have dilapidated infrastructure, unsanitary conditions and are overcrowded;
- Human and financial resource allocation to mental health is grossly inadequate.

### What this study adds

- A structured and comprehensive assessment on the quality of care and observance of human rights in a sub-Saharan African country;
- Recommendations for service transformation at facility and systems level that may be applicable across other sub-Saharan African countries.
